# Transportation to work by sexual orientation

**DOI:** 10.1371/journal.pone.0263687

**Published:** 2022-02-15

**Authors:** Sonia Oreffice, Dario Sansone

**Affiliations:** 1 Department of Economics, Business School, University of Exeter, Exeter, United Kingdom; 2 IZA, Bonn, Germany; 3 HCEO, Department of Economics, University of Chicago, Chicago, Illinois, United States of America; University of Luxembourg and Luxembourg Institute of Socio-Economic Research (LISER), LUXEMBOURG

## Abstract

We analyze differences in mode of transportation to work by sexual orientation, using the American Community Survey 2008–2019. Working individuals in same-sex couples are significantly less likely to drive to work than working men and women in different-sex couples. This gap is particularly stark among men: on average, almost 12 percentage point (or 13%) lower likelihood of driving to work for men in same-sex couples. Working individuals in same-sex couples are also more likely to use public transport, walk, or bike to work. Men and women are 7 and 3 percentage points more likely, respectively, to take public transportation to work than those in different-sex couples. Working men are also more likely to work from home–while working women are less likely–than those in different-sex couples. These differences persist after controlling for demographic characteristics, partner’s characteristics, location, fertility, marital status, occupation or industry, and family income. Additional evidence from the General Social Survey 2008–2018 suggests that these disparities by sexual orientation may be due to lesbian, gay, and bisexual individuals valuing the environment more than straight individuals.

## Introduction

There is by now a large and rising share (5.6%) of the US population who identifies as LGBT [1], with a large fraction of these sexual and gender minorities active in the labor market [2]. Meanwhile, there are increasing environmental and health concerns associated to passive modes of transportation to work, especially in the US where up to 88% of individuals drive to work [3]. The vast literature on transportation, demography, health, and the environment systematically reports that passive commuting to work–driving to work in particular–is associated with higher risk of diabetes, high blood pressure, high cholesterol, anxiety, depression, back pain, and cardiovascular diseases [4, 5], and that “vehicles are America’s biggest air quality compromisers, producing about one-third of all US air pollution” [6]. On the other hand, there is only scant anecdotal evidence that when it comes to the environment, LGBTQ+ adults are “greener” and more likely to express concerns about the environment than heterosexual adults.

Our main analysis estimates whether there are any differences in mode of transportation to work by sexual orientation, using recent data from the American Community Survey (ACS) 2008–2019 to compare working individuals in same-sex and different-sex couples, also by marital status and fertility. We highlight differences in driving to work (by auto, truck, van, or motorcycle), taking public transportation, walking or biking to work, and in working from home.

The ACS are annual cross-sectional data representative of the US population (a wave includes 1% of the US population), and provide the largest sample of detailed demographic, labor, and socio-economic information on individuals in same-sex couples, along with standard samples of individuals in different-sex couples. These data allow identification only of members of same-sex couples, but not of single LGBTQ+ individuals. Indeed, same-sex couples can be identified in the ACS by matching household heads with their same-sex spouses or same-sex unmarried partners. Previous research confirmed that the vast majority of individuals in same-sex couples in the ACS are indeed sexual minorities in a romantic relationship [2]. In addition to such information on couple type, we exploit the variable “Means of transportation to work”, reporting the primary means of transportation to work over the course of the week preceding the interview for all adults who worked in that week. In the ACS, this information is available for the respondent but also for their unmarried partner or spouse, if present and working the week preceding the interview. We build the most recent and largest sample with detailed demographic, labor, and transportation to work information on individuals in same-sex couples, along with a sample of individuals in different-sex couples, focusing on employed adults aged 18 to 64.

Our analysis is related with the transportation literature, where a gender commuting gap clearly emerges: not only women suffer more from commuting in terms of their physical and mental health [7, 8], but they make work travel choices that may penalize their job opportunities [9, 10]. Similarly, [11] use the Dutch Time Use Survey and estimate relevant gender differences between household responsibilities and women’s commuting behavior to work. The literature using time use data also shows that socio-demographic characteristics affect the choice of mode of transportation to work, and specifically of non-motorized alternatives, in the US and other countries [12]. Interestingly, [13] even estimate with American Time Use Survey data that immigrants coming from more gender-equal countries display smaller gender commuting gaps among parents in the US.

These studies reveal the relevant health and work outcomes at stake with transportation to work, and its prevalent gender disparity, along with reporting that health and well-being are greater with active or public transport work travel than driving to work. We believe that it is interesting to analyze mode of transportation to work beyond gender and specifically to consider sexual orientation (as proxied by being a member of a same-sex couple) as an additional determinant of work transportation patterns. Indeed, same-sex couples may be less subject to household specialization and work-family balance constraints than different-sex couples are, and/or may have different health and environment preferences.

The literature on same-sex couples has already highlighted both similarities and differences with respect to different-sex couples, also in terms of work patterns. For instance, [14] emphasized differences by couple type in biological constraints affecting fertility, location, household specialization, and human capital choices, while [15] discussed differences in field of study and occupational choices. [16] reported that same-sex couples are less likely to own a home than married different-sex ones. [17] showed that the specialization gap between same-sex and different-sex couples narrows across birth cohorts. [18] estimated that same-sex couples have similar labor supply responses to intra-household bargaining power to heterosexual couples. Overall, most studies find that lesbian women earn on average higher wages than heterosexual women, while gay men earn on average lower wages than heterosexual men, but some (not all) of these differences are decreasing over time [2].

In terms of same-sex couples and transportation, [19] used the American Time Use surveys from 2003 to 2012 to measure household-related travel choices of same-sex couples and different-sex couples. However, their sample contains only 133 men and 168 women in same-sex couples, it cannot distinguish between married and unmarried same-sex couples, and information on only one of the members of the couple is available. [20] measured private and public transportation differences among same-sex and different-sex couples using the ACS only from 2007 to 2011: however, same-sex couples cannot be reliably identified in the 2007 ACS data [21], while until 2012 married couples are reported as unmarried in the data. Therefore, they compare *unmarried same-sex* partners to *married different-sex* couples, potentially confounding sexual orientation differences with marital status and classification disparities. We analyze all recent modes of transportation to work, including working from home, and supplementary data, to explore marital patterns and additional potential mechanisms in terms of individual characteristics and couple roles.

We estimate significant differences by couple type in all work transportation arrangements, among working men and women, with the largest absolute difference reported for driving to work, and the smallest one for working from home. Working men and women in same-sex couples are less likely to drive to work than those in different-sex couples, but more likely to take public transportation or do active commuting such as walking or biking to work. The male disparity is always larger than the female one: the average gap represents a 13% lower likelihood of driving to work for working men in same-sex couples (4% for working women in same-sex couples), whereas for the other less popular work transportation arrangements the average gaps correspond to a higher likelihood of 69% or more. As to working from home, the average male couple difference corresponds to a higher likelihood of 45%. Our main empirical analysis controls for state and year fixed effects, the respondent’s age, education, race, ethnicity, their partner/spouse’s characteristics, as well as marital status and fertility. It is robust to controlling for occupation or industry, family income, to excluding students or military personnel, or to focusing on younger or older workers, household heads, or primary earners.

We then turn to the most recent General Social Survey (GSS) data, specifically the biannual waves from 2008 to 2018. In the GSS, gay, lesbian and bisexual individuals can be directly identified from survey questions on sexual orientation, although the sample size is notoriously very small for non-heterosexuals, yielding about 450 non-heterosexual individuals across all its biannual waves. However, a few questions about attitudes toward the environment were asked in multiple waves, such as how interested the respondent was in environmental pollution, whether they were concerned that the government spent too little on the environment, or on alternative energy sources. Our analysis of these three questions by sexual orientation in the GSS shows that lesbian, gay, and bisexual individuals have stronger environmental preferences than straight individuals (around 10% difference), suggesting that the estimated differences in transportation choices to work may be due to individuals in same-sex couples caring more about the environment than straight individuals.

We hope that this study may provide guidance to policymakers devising environmental and transportation policies aimed at reducing car usage, as well as at addressing inequalities, and health campaigns focusing on various communities in the US and abroad, but also to companies targeting healthier and productive work environments and schedules.

The paper is organized as follows. Section 2 describes the data and the empirical specification. Section 3 presents the empirical results. Section 4 concludes the paper.

## Materials and methods

### American community survey data

The main dataset used in our empirical analysis is the version of the ACS data publicly available through IPUMS-USA [22]. The ACS is a nationally-representative repeated cross-section that has been conducted every year since 2000 in the US. It contains demographic, economic, social, work and housing information. Since 2005, it has included a 1% random sample of the US population. Although there are no direct questions on sexual orientation, it is possible to identify individuals in same-sex couples living together. Indeed, household members can be classified as “unmarried partners” when recording their relationships to the household head. Importantly, roommates and unmarried partners are treated as two separated categories. Since 2012, same-sex couples have been allowed to report their actual marital status (between 2000 and 2012, same-sex married spouses were imputed as unmarried same-sex partners). S1 and S2 Tables report sample sizes by year, couple type, sex, and marital status.

Unmarried “heads” and “unmarried partners”, married “heads” and “spouses” were extracted from the ACS data using the variable “relationship to household head”. The household head is defined as the person who owns or rents the house, apartment, or mobile home (if there is no such person, the first person listed can be any adult living in the household). Using the variable “sex”, couples with the head and the unmarried partner (or the spouse) sharing the same sex were then classified as same-sex couples, and those of different sex as different-sex couples.

We use data until the latest available wave of 2019. We start from 2008 because the US Census Bureau implemented several changes between 2007 and 2008 to reduce the number of different-sex couples misclassified as same-sex (due to reporting errors in the sex question), which resulted in more reliable identification of same-sex couples [21]. We drop observations with imputed sex or relation to the household head to further reduce such measurement errors, following common practice in this literature [14]. Notwithstanding these issues, the US Census and the ACS remain the largest and most reliable data on same-sex couples [23].

## Methodology

We focus on employed adults aged 18 to 64 who worked the week before the survey. As previewed in the introduction, our main variable of interest is “Means of transportation to work”, reporting the primary means of transportation to work over the course of the week preceding the interview for all individuals who worked during that week. This information is available for the respondent but also for their unmarried partner or spouse, if present and working the week preceding the interview.

The following equation is estimated for each individual *i* living in state *s* at time *t*:

$$y_{ist}=\alpha +\beta SSC_{ist}+\delta_{s}+\mu_{t}+\gamma X_{ist}+\varepsilon_{ist}$$

The main empirical specification estimates a linear probability model where the dependent variable *y*_*ist*_ is a dummy variable corresponding to one of the four modes of transportation to work under consideration: driving to work, using public transport, biking or walking to work, or working from home. Most of the empirical analysis examines how a binary indicator for being in a same-sex couple (*SSC*_*ist*_) is associated to each of these types of transportation to work. The other main regressors are state and year fixed effects (*δ*_*S*_ and *μ*_*t*_), and the individual-level controls (*X*_*ist*_): the respondent’s age, race, ethnicity, and education, their partner/spouse’s characteristics, the couple’s marital status, and the number of own children living in the household. All variables used in the empirical analysis are described in S1 Text. In our sensitivity analysis, we also add a set of dummy variables for occupation or industry, as well as family income, to the controls *X*_*ist*_. Heteroskedasticity-robust standard errors are used throughout, as well as individual weights.

In our sample, men and women in same-sex couples are on average younger, more educated, more likely to be white, less likely to have children or be married, and more likely to be employed, than men and women in different-sex couples (S4 Table provides detailed summary statistics). This is in line with what previous literature on sexual orientation has documented in the US [2, 18].

### Additional evidence from the general social survey

As additional supporting evidence, we also use the most recent GSS data, specifically the biannual waves from 2008 to 2018. In the GSS, gay, lesbian and bisexual individuals can be directly identified from survey questions on sexual orientation. However, the sample size is small for non-heterosexuals: the dataset includes around 450 non-heterosexual individuals across all its biannual waves (S12 Table), of which only 288 are employed.

Nevertheless, a few questions about attitudes toward the environment were asked in multiple waves: whether the US was spending too much money, too little money, or about the right amount, on “the environment” and on “improving and protecting the environment”; on “developing alternative energy sources”; as well as whether the respondent was very interested, moderately interested or not at all interested in “issues about environmental pollution”. We test whether the answers to these three types of questions differ by sexual orientation (lesbian, gay, bisexual, versus straight). S1 Text provides more details on the GSS data and variables.

## Results

### Descriptive statistics

**Fig 1:**
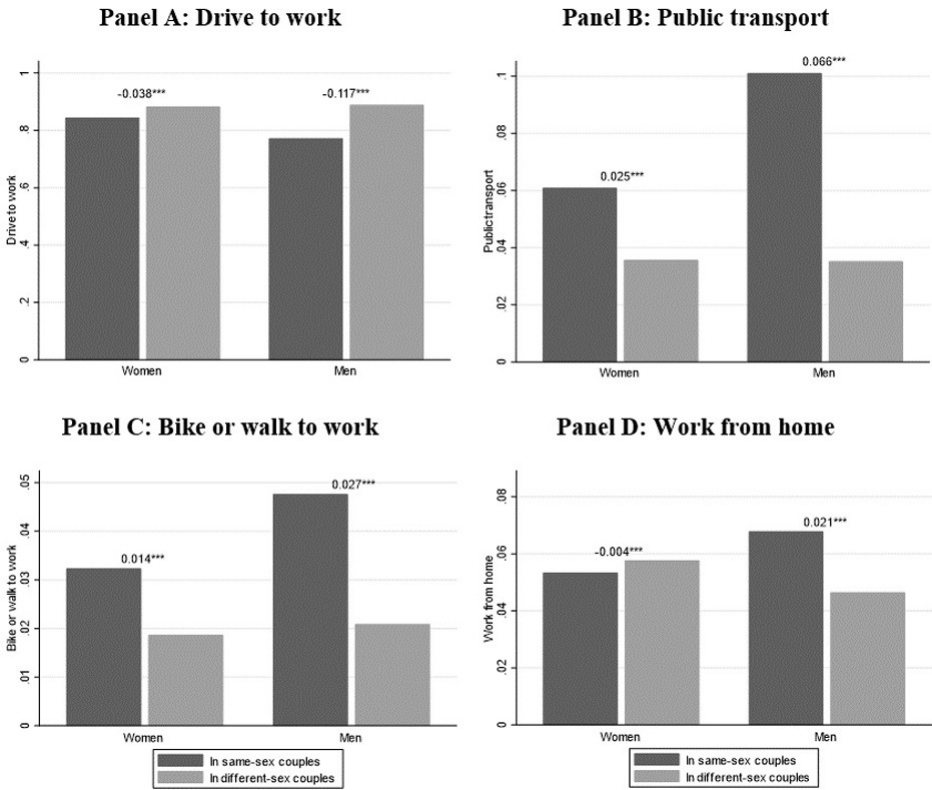
Means of transportation to work by sex and couple type. The number above each bar is the gap between the share of men or women in same-sex couples vs. in different-sex couples by mean of transportation. Weighted statistics. Source: ACS 2008–2019. * p < 0.10, ** p < 0.05, *** p < 0.01.

Fig 1 presents means of mode of transportation to work by couple type and sex. All the four work transportation arrangements exhibit significant differences by couple type, with the largest absolute difference reported for driving to work, and the smallest one for working from home. The disparity among working men in same-sex and different-sex couples is always larger than the one among working women, but they are all significant at the 1% level. S3 Table reports more detailed average comparisons for mode of transportation to work by sex and couple type.

### Multivariate analysis of driving to work

Table 1 reports the main regression results of driving to work on a binary indicator for being in a same-sex couple, separately for working women (Panel A) and working men (Panel B). Starting from the basic correlation in Column 1, controls are incrementally added, from state and year fixed effects (Column 2), to the respondent’s age, race, ethnicity, and education (Column 3), their partner/spouse’s characteristics (Column 4), their marital status and the number of own children living in the household (Column 5).

**Table 1:** Differences in driving to work by sex and couple type.

	(1)	(2)	(3)	(4)	(5)
*Panel A*: *Women in SSC and DSC*					
In a same-sex couple	-0.038***	-0.030***	-0.030***	-0.027***	-0.020***
	(0.002)	(0.002)	(0.002)	(0.002)	(0.002)
Observations	4,411,409	4,411,409	4,411,409	4,411,409	4,411,409
Mean of dependent variable	0.881	0.881	0.881	0.881	0.881
R^2^	0.000	0.040	0.044	0.046	0.047
*Panel B*: *Men in SSC and DSC*					
In a same-sex couple	-0.117***	-0.099***	-0.091***	-0.091***	-0.072***
	(0.002)	(0.002)	(0.002)	(0.002)	(0.002)
Observations	5,210,836	5,210,836	5,210,836	5,210,836	5,210,836
Mean of dependent variable	0.887	0.887	0.887	0.887	0.887
R^2^	0.002	0.041	0.050	0.051	0.052
*Controls for*:					
State and year FE		✓	✓	✓	✓
Demographic controls			✓	✓	✓
Partner/spouse controls				✓	✓
Fertility and marital status					✓

“SSC” indicates same-sex couples, “DSC” indicates different-sex couples. Heteroskedasticity-robust standard errors in parentheses. Weighted regressions and statistics. Respondents younger than 18 or older than 64 have been excluded. *Demographic controls* include respondent’s age, race, ethnicity, and education. *Partner/spouse controls* include spouse’s or unmarried partner’s age, race, ethnicity, and education. *Fertility* includes the number of own children (of any age or marital status) residing with the respondent, as well as the number of own children age 4 and under residing with the respondent. All variables are described in detail in S1 Text. Source: ACS 2008–2019.

* *p* < 0.10,

** *p* < 0.05,

*** *p* < 0.01.

On average, between 88% and 89% of our sample drives to work, so that the almost 4 percentage point reduction in the likelihood of driving to work among working women in same-sex couples (Column 1) represents a 4% reduction with respect to working women in different-sex couples. Even more striking are the disparities among working men: three times as large as those among women. The estimated coefficients indicate a reduction of 12 percentage points in the likelihood of driving to work among working men in same-sex couples, corresponding to 13% lower propensity to drive to work with respect to working men in different-sex couples.

These sizable gaps represent important environmental and health gains. In our 2008–2019 sample of 5.2 million partnered working men, on average 4.6 million drove to work: therefore, if men in different-sex couples had driven to work on average as frequently as men in same-sex couples, a 12 percentage point decline would have been equivalent to over 0.6 million fewer men driving. If we extrapolate the same reasoning to the 2019 ACS population means, this reduction is equivalent to over 1 million women and almost 5 million men not driving to work in a year, hugely cutting CO_2_ emissions (around 50 million tons/year), The average US passenger vehicle releases 650g of CO_2_/km [29]. The average American drives 16 miles to work each way [30]. Assuming a US average of 250 workdays/year, 6 million people not driving to work would reduce CO_2_ emissions by around 50 million tons/year. For comparison, US annual per capita CO_2_ emission in 2018 was 15 tons [31]. negative externalities, and improving health.

All these gaps are significant at the 1% level, and robust to controlling for demographic characteristics, partner’s characteristics, fertility, and marital status, although their magnitude decreases from columns 2 to 5 (the estimated coefficients of the same-sex couple indicator are statistically different between the first and the last columns). In Table 2, we include additional controls and show that controlling for student status, being in the military, or including occupation or industry fixed effects does not substantially affect the estimated coefficient for same-sex couples: thus, these negative and significant differences cannot simply be attributed to different choices of jobs or workplace locations by workers in same-sex couples. The last column of Table 2 adds family income as regressor, showing that the estimated differences for same-sex couples are essentially the same as those in column 5 of Table 1.

**Table 2:** Differences in driving to work by sex and couple type. Additional controls.

	(1)	(2)	(3)	(4)
*Panel A*: *Women in SSC and DSC*				
In a same-sex couple	-0.020***	-0.019***	-0.018***	-0.021***
	(0.002)	(0.002)	(0.002)	(0.002)
Observations	4,411,409	4,411,409	4,411,409	4,411,409
Mean of dependent variable	0.881	0.881	0.881	0.881
R^2^	0.047	0.099	0.098	0.047
*Panel B*: *Men in SSC and DSC*				
In a same-sex couple	-0.072***	-0.062***	-0.062***	-0.071***
	(0.002)	(0.002)	(0.002)	(0.002)
Observations	5,210,836	5,210,836	5,210,836	5,210,836
Mean of dependent variable	0.887	0.887	0.887	0.887
R^2^	0.053	0.088	0.088	0.053
*Controls for*:				
State and year FE	✓	✓	✓	✓
Demographic controls	✓	✓	✓	✓
Partner/spouse controls	✓	✓	✓	✓
Fertility and marital status	✓	✓	✓	✓
Student and army status	✓	✓	✓	✓
Occupation FE		✓		
Industry FE			✓	
Family income				✓

See also notes in Table 1. Source: ACS 2008–2019.

* *p* < 0.10,

** *p* < 0.05,

*** *p* < 0.01.

A battery of robustness checks (S5–S10 Tables) confirms that same-sex couples exhibit a lower propensity to drive to work. These include clustering SE, not using weights, using a logit rather than a linear probability model, restricting the regression sample to household heads, partners/spouses, main earner in the couple, individuals whose partner/spouse works as well, individuals aged 40 or younger, individuals older than 40, or the 2012–2019 ACS samples. In addition, disentangling our estimates by race and ethnicity (S7 Table) shows the same pattern of driving to work by couple type for Whites and Blacks, whereas among Asians and Hispanics only the male transport gap is significant.

Table 3 illustrates these same disparities in driving to work by marriage, parenthood, whether couples live in the city center, or for full-time workers. These estimates reveal that the largest gaps for women come from married couples without children. The disparity by couple type is always larger among men, also when couples live in the city center [24], while full-time workers exhibit the same gaps by type of couple and sex as in the full sample. Furthermore, the estimates for married women or men with children in Column 1 are statistically different from those for married women or men without children in Column 2. Similarly, the estimates for unmarried women or men with children in Column 3 are statistically different from those for unmarried women or men without children in Column 4 (although for women the difference between the two estimates is only significant at the 5-percent level). This additional evidence suggest that these distinctive patterns cannot be explained by sexual minority men’s preference to live in city centers, or by freelance/part-time workers. These estimates also reflect households/couples’ decisions related to fertility: in the presence of children, couples are more similar in terms of driving to work.

**Table 3:** Differences in driving to work by sex and couple type. Sub-sample analysis.

	Married w/children	Married w/o children	Unmarried w/children	Unmarried w/o children	City center	Only full-time workers
	(1)	(2)	(3)	(4)	(5)	(6)
*Panel A*: *Women in SSC and DSC*						
In a same-sex couple	-0.024***	-0.062***	-0.002	0.010***	0.003	-0.020***
	(0.005)	(0.004)	(0.005)	(0.003)	(0.005)	(0.002)
Observations	1,518,968	1,049,278	144,190	227,662	452,789	2,923,255
Mean of dependent variable	0.880	0.874	0.893	0.853	0.710	0.891
R^2^	0.040	0.051	0.055	0.096	0.234	0.054
*Panel B*: *Men in SSC and DSC*						
In a same-sex couple	-0.049***	-0.075***	-0.017*	-0.059***	-0.067***	-0.075***
	(0.007)	(0.004)	(0.010)	(0.003)	(0.004)	(0.002)
Observations	1,972,381	1,092,622	166,510	235,897	547,612	4,658,202
Mean of dependent variable	0.891	0.876	0.897	0.841	0.739	0.893
R^2^	0.049	0.056	0.048	0.102	0.216	0.053
*Controls for*:						
State and year FE	✓	✓	✓	✓	✓	✓
Demographic controls	✓	✓	✓	✓	✓	✓
Partner/spouse controls	✓	✓	✓	✓	✓	✓
Fertility and marital status					✓	✓

See also notes in Table 1. Source: ACS 2012–2019 in Columns 1–4; 2008–2019 in Column 5.

* *p* < 0.10,

** *p* < 0.05,

*** *p* < 0.01.

### Multivariate analysis of other modes of transportation to work

We then turn to examine differences by couple type in the other means of transportation to work: in Tables 4 and 5 we present differences by couple type in active commuting such as walking or biking to work, in Tables 6 and 7 we focus on the probability of using public transportation to work, while in Tables 8 and 9 we estimate the differences in working from home.

**Table 4:** Differences in biking or walking to work by sex and couple type.

	(1)	(2)	(3)	(4)	(5)
*Panel A*: *Women in SSC and DSC*					
In a same-sex couple	0.014***	0.013***	0.012***	0.012***	0.006***
	(0.001)	(0.001)	(0.001)	(0.001)	(0.001)
Observations	4,411,409	4,411,409	4,411,409	4,411,409	4,411,409
Mean of dependent variable	0.019	0.019	0.019	0.019	0.019
Adjusted R^2^	0.000	0.008	0.009	0.009	0.010
*Panel B*: *Men in SSC and DSC*					
In a same-sex couple	0.027***	0.023***	0.022***	0.022***	0.013***
	(0.001)	(0.001)	(0.001)	(0.001)	(0.001)
Observations	5,210,836	5,210,836	5,210,836	5,210,836	5,210,836
Mean of dependent variable	0.021	0.021	0.021	0.021	0.021
Adjusted R^2^	0.000	0.008	0.009	0.009	0.010
*Controls for*:					
State and year FE		✓	✓	✓	✓
Demographic controls			✓	✓	✓
Partner/spouse controls				✓	✓
Fertility and marital status					✓

See also notes in Table 1. Source: ACS 2008–2019.

* *p* < 0.10,

** *p* < 0.05,

*** *p* < 0.01.

**Table 5:** Differences in biking or walking to work by sex and couple type. Additional controls.

	(1)	(2)	(3)	(4)
*Panel A*: *Women in SSC and DSC*				
In a same-sex couple	0.006***	0.007***	0.007***	0.006***
	(0.001)	(0.001)	(0.001)	(0.001)
Observations	4,411,409	4,411,409	4,411,409	4,411,409
Mean of dependent variable	0.019	0.019	0.019	0.019
R^2^	0.010	0.017	0.015	0.010
*Panel B*: *Men in SSC and DSC*				
In a same-sex couple	0.013***	0.010***	0.009***	0.013***
	(0.001)	(0.001)	(0.001)	(0.001)
Observations	5,210,836	5,210,836	5,210,836	5,210,836
Mean of dependent variable	0.021	0.021	0.021	0.021
R^2^	0.010	0.022	0.021	0.011
*Controls for*:				
State and year FE	✓	✓	✓	✓
Demographic controls	✓	✓	✓	✓
Partner/spouse controls	✓	✓	✓	✓
Fertility and marital status	✓	✓	✓	✓
Student and army status	✓	✓	✓	✓
Occupation FE		✓		
Industry FE			✓	
Family income				✓

See also notes in Table 1. Source: ACS 2008–2019.

* *p* < 0.10,

** *p* < 0.05,

*** *p* < 0.01.

**Table 6:** Differences in taking public transport to work by sex and couple type.

	(1)	(2)	(3)	(4)	(5)
*Panel A*: *Women in SSC and DSC*					
In a same-sex couple	0.025***	0.021***	0.022***	0.021***	0.011***
	(0.001)	(0.001)	(0.001)	(0.001)	(0.001)
Observations	4,411,409	4,411,409	4,411,409	4,411,409	4,411,409
Mean of dependent variable	0.036	0.036	0.036	0.036	0.036
Adjusted R^2^	0.000	0.078	0.091	0.092	0.095
*Panel B*: *Men in SSC and DSC*					
In a same-sex couple	0.066***	0.054***	0.051***	0.052***	0.043***
	(0.001)	(0.001)	(0.001)	(0.001)	(0.001)
Observations	5,210,836	5,210,836	5,210,836	5,210,836	5,210,836
Mean of dependent variable	0.036	0.036	0.036	0.036	0.036
Adjusted R^2^	0.001	0.081	0.092	0.093	0.094
*Controls for*:					
State and year FE		✓	✓	✓	✓
Demographic controls			✓	✓	✓
Partner/spouse controls				✓	✓
Fertility and marital status					✓

See also notes in Table 1. Source: ACS 2008–2019.

* *p* < 0.10,

** *p* < 0.05,

*** *p* < 0.01.

**Table 7:** Differences in taking public transport to work by sex and couple type. Additional controls.

	(1)	(2)	(3)	(4)
*Panel A*: *Women in SSC and DSC*				
In a same-sex couple	0.011***	0.011***	0.010***	0.011***
	(0.001)	(0.001)	(0.001)	(0.001)
Observations	4,411,409	4,411,409	4,411,409	4,411,409
Mean of dependent variable	0.036	0.036	0.036	0.036
R^2^	0.095	0.104	0.106	0.095
*Panel B*: *Men in SSC and DSC*				
In a same-sex couple	0.043***	0.039***	0.038***	0.042***
	(0.001)	(0.001)	(0.001)	(0.001)
Observations	5,210,836	5,210,836	5,210,836	5,210,836
Mean of dependent variable	0.036	0.036	0.036	0.036
R^2^	0.094	0.108	0.109	0.095
*Controls for*:				
State and year FE	✓	✓	✓	✓
Demographic controls	✓	✓	✓	✓
Partner/spouse controls	✓	✓	✓	✓
Fertility and marital status	✓	✓	✓	✓
Student and army status	✓	✓	✓	✓
Occupation FE		✓		
Industry FE			✓	
Family income				✓

See also notes in Table 1. Source: ACS 2008–2019.

* *p* < 0.10,

** *p* < 0.05,

*** *p* < 0.01.

**Table 8:** Differences in working from home by sex and couple type.

	(1)	(2)	(3)	(4)	(5)
*Panel A*: *Women in SSC and DSC*					
In a same-sex couple	-0.004***	-0.007***	-0.008***	-0.010***	0.0004
	(0.001)	(0.001)	(0.001)	(0.001)	(0.0010)
Observations	4,411,409	4,411,409	4,411,409	4,411,409	4,411,409
Mean of dependent variable	0.058	0.058	0.058	0.058	0.058
Adjusted R^2^	0.000	0.004	0.009	0.011	0.014
*Panel B*: *Men in SSC and DSC*					
In a same-sex couple	0.021***	0.019***	0.015***	0.014***	0.015***
	(0.001)	(0.001)	(0.001)	(0.001)	(0.001)
Observations	5,210,836	5,210,836	5,210,836	5,210,836	5,210,836
Mean of dependent variable	0.047	0.047	0.047	0.047	0.047
Adjusted R^2^	0.000	0.002	0.012	0.013	0.013
*Controls for*:					
State and year FE		✓	✓	✓	✓
Demographic controls			✓	✓	✓
Partner/spouse controls				✓	✓
Fertility and marital status					✓

See also notes in Table 1. Source: ACS 2008–2019.

* *p* < 0.10,

** *p* < 0.05,

*** *p* < 0.01.

**Table 9:** Differences in working from home by sex and couple type. Additional controls.

	(1)	(2)	(3)	(4)
*Panel A*: *Women in SSC and DSC*				
In a same-sex couple	0.001	-0.001	-0.001	0.001
	(0.001)	(0.001)	(0.001)	(0.001)
Observations	4,411,409	4,411,409	4,411,409	4,411,409
Mean of dependent variable	0.058	0.058	0.058	0.058
R^2^	0.014	0.082	0.085	0.015
*Panel B*: *Men in SSC and DSC*				
In a same-sex couple	0.016***	0.011***	0.014***	0.015***
	(0.001)	(0.001)	(0.001)	(0.001)
Observations	5,210,836	5,210,836	5,210,836	5,210,836
Mean of dependent variable	0.047	0.047	0.047	0.047
R^2^	0.013	0.055	0.053	0.013
*Controls for*:				
State and year FE	✓	✓	✓	✓
Demographic controls	✓	✓	✓	✓
Partner/spouse controls	✓	✓	✓	✓
Fertility and marital status	✓	✓	✓	✓
Student and army status	✓	✓	✓	✓
Occupation FE		✓		
Industry FE			✓	
Family income				✓

See also notes in Table 1. Source: ACS 2008–2019.

* *p* < 0.10,

** *p* < 0.05,

*** *p* < 0.01.

Working individuals in same-sex couples are more likely to walk or bike to work than individuals in different-sex couples, with twice as large differences among men than among women, all significant at the 1% level across specifications (Table 4). Table 5 illustrates that controlling for student status, being in the military, including occupation or industry fixed effects, or family income, does not affect the estimated coefficient for same-sex couples.

The same pattern holds for public transportation in Tables 6 and 7: working individuals in same-sex couples are more likely to take it to work, the difference by sexual orientation is around three times as large for men than for women, and controlling for student status, being in the military, occupation or industry fixed effects, or family income is immaterial to the estimated coefficient of the same-sex couple indicator.

In terms of magnitude, on average, 4% of individuals in the US take public transport to work, so that a difference in around 3 percentage points among women, and 7 percentage points among men, represent a 69% and 183% increase with respect to those in different-sex couples. For biking or walking to work, the average in the US population is 2%, so that the difference in 1 percentage point among women, and 3 percentage points among men, represent a 74% and 129% increase, respectively. S11 Table presents multinomial logit regressions of type of work transport, confirming our main findings: individuals in same-sex couples are more likely to use public transportation or to walk or bike than driving to work (women almost twice as likely and men three times).

Tables 8 and 9 present the estimated differences by couple type in working from home. Working men in same-sex couples are more likely to work from home than working men in different-sex couples, whereas working women in same-sex couples are less likely to work from home than those in different-sex couples. The size of the gap is much larger for men, representing almost a 50% increase with respect to men in different-sex couples, out of the 5% that on average work from home, whereas among women, 6% on average work from home, and the sexual orientation gap corresponds at most to a 1% decrease, which actually turns to insignificant among women when estimated with additional controls (Table 9, and last column of Table 8).

### Evidence on environmental preferences

This evidence highlights that work transportation differences between same-sex and different-sex couples are still persistent nowadays among working men and women, and cannot be fully explained by factors such as age, race, education, marital status, presence of children in the household, occupation, industry, family income, or by focusing on those living in city centers, or on primary earners. Similarly, [20] found that choice of transportation mode was not affected by living in a gay or lesbian neighborhood.

We suggest that the more environmentally-conscious choices of transportation to work that we document for same-sex couples may reflect that sexual minorities care more about the environment: indeed, the literature on transportation also emphasizes the massive negative impact on the environment of commuting to work by car (6). We investigate this explanation using a different data set, the GSS and its most recent biannual waves from 2008 to 2018. Gay, lesbian and bisexual individuals can be identified from survey questions on sexual orientation, while a few questions about the environment were asked in multiple waves, such as how interested the respondent was in environmental pollution, whether they were concerned that the government spent too little on the environment, or on alternative energy sources.

**Fig 2:**
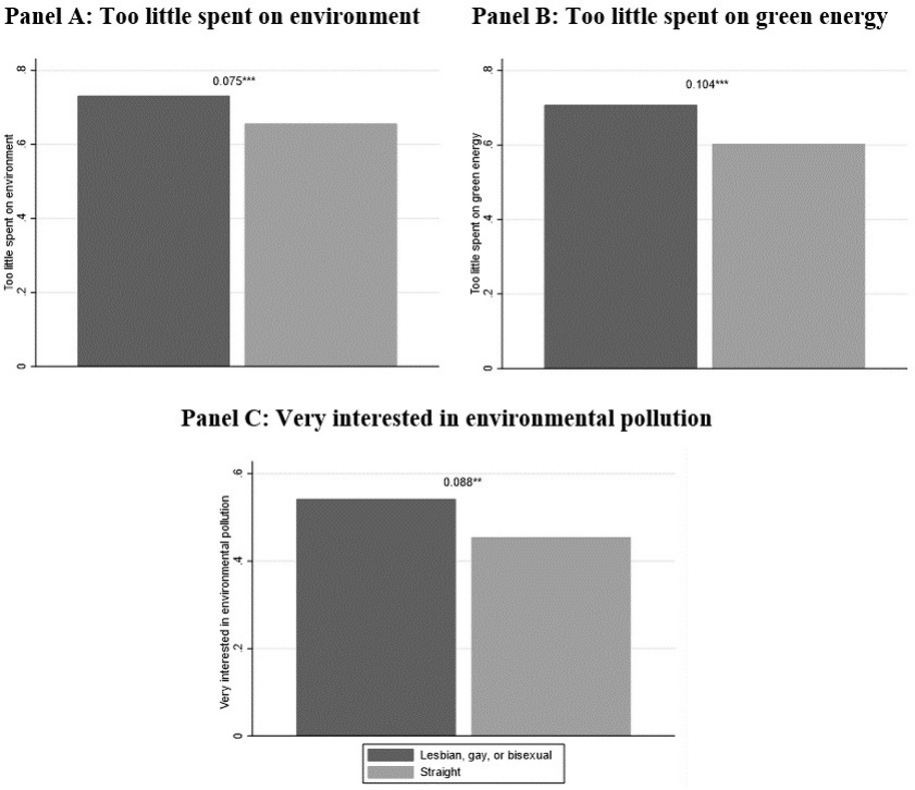
Environmental preferences by sexual orientation. The number above each bar is the difference by sexual orientation of the share of respondents who have a certain environmental preference. Weighted statistics. Source: GSS 2008–2018. * p < 0.10, ** p < 0.05, *** p < 0.01.

Fig 2 shows that there are significant differences by sexual orientation in attitudes toward the environment: lesbian, gay, and bisexual individuals hold more environmentally-friendly views and are more concerned about the environment than straight individuals. Indeed, lesbian, gay, and bisexual individuals are 8 percentage points more likely to believe that the US was spending too little on protecting the environment, 10 percentage points more likely to believe that the US was spending too little on developing alternative energy sources, and 9 percentage points more likely to report being very interested on issues about environmental pollution. We reach the same conclusion if we restrict our analysis to employed individuals (288 observations). In addition, S13 Table reports extended average comparisons for environmental preferences by sexual orientation.

## Discussion

This paper shows that employed individuals in same-sex couples are significantly less likely to drive to work than working men and women in different-sex couples, while they are more likely to use public transport, walk, or bike to work. These estimates represent significant and sizable differences by sexual orientation in all work traveling patterns. Gaps among men are larger than among women, and men in same-sex couples are also more likely to work from home. Additional analysis suggests that lesbian, gay, and bisexual individuals may value the environment more, leading them to choose alternative transportation modes to driving to work.

We believe that these findings represent an interesting addition to the literature on transportation to work. This literature emphasizes two main distinctive features: first, that mode of transportation, and driving to work in particular, affects an individuals’ health; second, that health outcomes and labor market outcomes associated to different transportation to work and commuting patterns are starkly different by gender. We go one step further to document that gender differences are more nuanced: when we take into account the sexual orientation of partnered or married men, and of partnered or married women, we find significant differences by couple type both among men and among women.

These work transportation choices made by individuals in same-sex households have direct health and environmental implications. Our study reveals that working men and women in same-sex couples make healthier and more environmentally conscious transportation to work choices than comparable men and women in different-sex couples: this could help policy makers and health practitioners to address transportation to work in a more diverse and less traditional family-oriented manner.

Gender norms affecting many household and work choices of different-sex couples may also play a role in the choices regarding mode of transportation to work, which in turn affect people’s health, the environment, and work opportunities of men and women. It may be possible to nudge people into making healthier and more environmentally friendly work transportation choices by weakening traditional gender norms among different-sex couples and enhancing diversity and gender inclusivity. (Public) transportation is related to gender and women’s safety [25]. Recent evidence from developing countries also indicates that mode of transportation is related to gender and human capital accumulation. For instance, the safety of transportation affects women’s human capital attainment in India in terms of college quality [26], while providing free bicycles increases girls’ school presence in rural Zambia and girls’ secondary school enrollment in India by reducing the time and safety cost of transportation [27, 28]. Estimating the work transportation differences among same-sex and different-sex couples may help to better understand the transportation decisions of men and women overall, and the factors that influence them.

We acknowledge our study’s limitations inherent to the ACS data: only LGBTQ+ individuals in same-sex partnerships or marriages can be identified. Single LGBTQ+ individuals are untraceable. Sexual orientation is instead available in the GSS, albeit associated to a small sample size. Finally, the lack of gender identity data precludes analyzing mode of transportation differences between transgender and cisgender individuals.

## Supporting information

S1 Text Variable description. (DOCX)

S1 Table ACS sample sizes. Individuals age 18–64 in same-sex and different-sex couples.
(DOCX)

S2 Table ACS sample sizes. Individuals age 18–64 in married and unmarried couples.
(DOCX)

S3 Table Mean comparisons for mode of transportation to work by sex and couple type. (DOCX)

S4 Table Descriptive statistics by sex and couple type. (DOCX)

S5 Table Drive to work. By sex and couple type. Additional restrictions.
(DOCX)

S6 Table Drive to work. By sex, couple type, and age group.
(DOCX)

S7 Table Drive to work. By sex, couple type, race, and ethnicity.
(DOCX)

S8 Table Drive to work. By sex, couple type, marital status, and fertility.
(DOCX)

S9 Table Drive to work. By sex, couple type, and position in household.
(DOCX)

S10 Table Drive to work. By sex and couple type. Additional subsamples.
(DOCX)

S11 Table Multinomial logit of type of transport to work (driving, public, or active). By sex and couple type.
(DOCX)

S12 Table GSS sample sizes. Individuals age 18–64 by sexual orientation.
(DOCX)

S13 Table Mean comparisons for environmental preferences by sexual orientation. (DOCX)

## References

[1] Jones JM. LGBT Identification Rises to 5.6% in Latest U.S. Estimate. 2021.

[2] Badgett MVL, Carpenter CS, Sansone D. LGBTQ Economics. J Econ Perspect. 2021;35: 141–170.

[3] McKenzie B. Who Drives to Work? Commuting by Automobile in the United States: 2013. Washington, D.C.; 2015.

[4] Hoehner CM, Barlow CE, Allen P, Schootman M. Commuting Distance, Cardiorespiratory Fitness, and Metabolic Risk. Am J Prev Med. 2012;42: 571–578. doi: 10.1016/j.amepre.2012.02.020 22608372 PMC3360418

[5] Kylstra C. 10 Things Your Commute Does to Your Body. Time. Feb 2014.

[6] National Geographic. The environmental impacts of cars, explained. National Geographic. Sep 2019: 1–5.

[7] Roberts J, Hodgson R, Dolan P. “It’s driving her mad”: Gender differences in the effects of commuting on psychological health. J Health Econ. 2011;30: 1064–1076. doi: 10.1016/j.jhealeco.2011.07.006 21855154

[8] Künn-Nelen A. Does Commuting Affect Health? Health Econ. 2016;25: 984–1004. doi: 10.1002/hec.3199 26010157

[9] Black DA, Kolesnikova N, Taylor LJ. Why do so few women work in New York (and so many in Minneapolis)? Labor supply of married women across US cities. J Urban Econ. 2014;79: 59–71.

[10] Barbanchon T Le, Rathelot R, Roulet A. Gender Differences in Job Search: Trading off Commute against Wage. Q J Econ. 2021;136: 381–426.

[11] Gimenez-Nadal JI, Molina JA. Commuting time and household responsibilities: Evidence using propensity score matching. J Reg Sci. 2016;56: 332–359.

[12] Molina JA, Ignacio Giménez-Nadal J, Velilla J. Sustainable Commuting: Results from a Social Approach and International Evidence on Carpooling. Sustainability. 2020;12: 9587.

[13] Marcén M, Morales M. Culture and the cross-country differences in the gender commuting gap. J Transp Geogr. 2021;96: 103184.

[14] Black DA, Sanders SG, Taylor LJ. The economics of lesbian and gay families. J Econ Perspect. 2007;21: 53–70.

[15] Sansone D, Carpenter CS. Turing’s Children: Representation of Sexual Minorities in STEM. PLoS One. 2020;15: e0241596. doi: 10.1371/journal.pone.0241596 33206668 PMC7673532

[16] Jepsen CA, Jepsen LK. Does home ownership vary by sexual orientation? Reg Sci Urban Econ. 2009;39: 307–315.

[17] Giddings L, Nunley JM, Schneebaum A, Zietz J. Birth Cohort and the Specialization Gap Between Same-Sex and Different-Sex Couples. Demography. 2014;51: 509–534. doi: 10.1007/s13524-013-0267-4 24585040

[18] Oreffice S. Sexual orientation and household decision making. Same-sex couples’ balance of power and labor supply choices. Labour Econ. 2011;18: 145–158.

[19] Smart MJ, Brown A, Taylor BD. Sex or sexuality? Analyzing the division of labor and travel in gay, lesbian, and straight households. Travel Behav Soc. 2017;6: 75–82.

[20] Klein NJ, Smart MJ. Travel mode choice among same-sex couples. Transp Res Part A Policy Pract. 2016;90: 1–13.

[21] U.S. Census. Frequently Asked Questions About Same-Sex Couple Households. US Census. 2013;August: 1–4.

[22] Ruggles S, Flood S, Foster S, Goeken R, Pacas J, Schouweiler M, et al. IPUMS USA: Version 11.0 [dataset]. Minneapolis, MN; 2021. Available: 10.18128/D010.V11.0.

[23] Sansone D. Pink work: Same-sex marriage, employment and discrimination. J Public Econ. 2019;180: 104086.

[24] Black DA, Gates G, Sanders S, Taylor L. Why Do Gay Men Live in San Francisco? J Urban Econ. 2002;51: 54–76.

[25] Zhen S. Rethinking public transportation for women’s safety and security. Bonn, Germany; 2021.

[26] Borker G. Safety First: Perceived Risk of Street Harassment and Educational Choices of Women. Work Pap. 2020.

[27] Muralidharan K, Prakash N. Cycling to School: Increasing Secondary School Enrollment for Girls in India. Am Econ J Appl Econ. 2017;9: 321–50.

[28] Fiala N, Narula K, Prakash N. Wheels of Change: Transforming Girl’s Lives with Bicycles. Work Pap. 2021.

[29] Timperley J. How our daily travel harms the planet. 2020.

[30] Harris D. How Far Do Americans Drive to Work on Average? 2007.

[31] World Bank. CO2 emissions (metric tons per capita). Washington, D.C.; 2018.

