# Supplementary material for: Transportation to work by sexual orientation
Source: PLoS One. 2022 Feb 15;17(2):e0263687. doi: 10.1371/journal.pone.0263687 (PMC8846529; doi:10.1371/journal.pone.0263687)
Supplement: S1 Text — (DOCX) [file pone.0263687.s001.docx]

**S1 Text. Variable description**

**S1.1 ACS Variables**

**S1.1.1 Dependent variables: Means of transportation to work**

Individuals were asked the following question:

*How did this person usually get to work LAST WEEK? If this person usually used more than one method of transportation during the trip, mark (X) the box of the one used for most of the distance.*

[ ] *Car, truck, or van*[ ] *Bus or trolley bus*[ ] *Streetcar or trolley car*[ ] *Subway or elevated*[ ] *Railroad*[ ] *Ferryboat*[ ] *Taxicab*[ ] *Motorcycle*[ ] *Bicycle*[ ] *Walked*[ ] *Worked at home -> SKIP to question 40a*[ ] *Other method*

From the answers to this question, a series of indicator variables has been constructed to record a person’s main mean of transportation to work. The indicator *drive to work* includes the options “car, truck, or van” and “motorcycle”.

The indicator *public transport* includes the options “bus or trolley bus”; “streetcar or trolley car”; “subway or elevated”; “railroad”; and “ferryboat”. It is worth noting that a few changes were implemented in the 2019 ACS survey: the option “bus or trolley bus” was relabelled “bus”; the option “subway or elevated” was relabelled “subway or elevated rail”; the option “railroad” was relabelled “long-distance train or commuter train”; the option “light rail, streetcar, or trolley” replaced “streetcar or trolley car”. Even if these changes may affect the exact option selected by an individual, they are highly unlikely to affect the proportion of individuals reporting public transport as their main mean of transportation, which is the dependent variable used in the empirical analysis.

This question on means of transportation to work was asked to all individuals age 16 or more who worked in the week preceding the interview. The aforementioned indicators have been coded as missing for individuals not working in the week preceding the interview.

**S1.1.2 Key independent variable: In a same-sex couple**

The ACS does not directly ask individuals about their sexual orientation. However, the ACS identifies a primary reference person, defined as “the person living or staying here in whose name this house or apartment is owned, being bought, or rented”. The ACS also collects information on the relationship to the primary reference person for all members of the household, and the range of possible relationships includes husband, wife, and unmarried partner (as a different category than roommate or other nonrelative). By combining such information, it has been possible to create an indicator variable equal to one if an individual was in a same-sex couple; zero if an individual was in a different-sex couple. Both individuals married to a same-sex spouse and individuals living with a same-sex unmarried partner have been coded as individuals in same-sex couples.

It is worth nothing that, in order to reduce measurement error, in 2019 the ACS survey question explicitly distinguished between “opposite-sex husband/wife/spouse”, “opposite-sex unmarried partner”, “same-sex husband/wife/spouse”, and “same-sex unmarried partner”. In addition, the options for unmarried partners were moved higher in the list of potential relation categories, thus increasing its salience.

**S1.1.3 Additional variables**

*Sex* reports whether the person was male or female. Note that sex in the ACS is reported as a binary variable.

*Age* reports a person’s age in years at the time of the interview. A similar variable has been constructed to report the age of a person’s spouse or unmarried partner.

*Race* includes a series of indicator variables constructed to record a person’s race: White, Black, Asian, or other races. The indicator *Asian* includes Chinese, Japanese, Other Asian or Pacific Islander. The indicator *other races* includes American Indian, Alaska Native, other race not listed, or individuals who selected two or three major races. A similar set of variables has been constructed to report the race of a person’s spouse or unmarried partner.

*Hispanic* is an indicator equal to one if a person self-identified as Mexican, Puerto Rican, Cuban, or Other Hispanic; zero otherwise. A similar variable has been constructed to report the ethnicity of a person’s spouse or unmarried partner.

*Higher Education* is an indicator equal to one if a person’s highest degree completed was a Bachelor’s degree or higher (Master’s degree, Professional degree beyond a Bachelor’s degree, Doctoral degree); zero otherwise. A similar variable has been constructed to report the education level of a person’s spouse or unmarried partner.

*Number of children* reports the number of own children (of any age or marital status) residing with each individual. This variable includes step-children and adopted children as well as biological children. This variable is coded as zero for people with no children present in the household.

*Number of children under age 5* reports the number of own children age 4 or under residing with each individual. This variable includes step-children and adopted children as well as biological children. This variable is coded as zero for people with no children under 5 present in the household.

*Married* is an indicator equal to one if a person is a member of a (same-sex or different-sex) married couple; zero otherwise.

*Student status* is an indicator equal to one if a person attended school or college in the 3 months preceding the interview; zero otherwise.

*In the army* is an indicator equal to one if a person reported being employed in the Armed forces (including “Armed forces: at work” and “Armed forces: with job but not at work”); zero otherwise.

*Employed* is an indicator equal to one if a person was working in the week preceding the interview; zero otherwise.

*In the labor force* is an indicator equal to one if a person was a part of the labor force, either working or seeking work, in the week preceding the interview; zero if a person was out of the labor force, or did not have a job, was looking for a job, but had not yet found one at the time of the interview.

*Total family income* reports the total pre-tax money income earned by one's family from all sources for the 12 months preceding the interview. Amounts are expressed in contemporary dollars, and not adjusted for inflation.

*Occupation* records a person’s primary occupation using the IPUMS harmonized occupation coding based on the Census Bureau's 2010 ACS occupation classification scheme. Unemployed persons were to give their most recent occupation, if they had worked in the 5 years preceding the interview, otherwise they were classified as “Unemployed, with No Work Experience in the Last 5 Years or Earlier or Never Worked”.

*Industry* reports the type of industry in which the person performed an occupation using the IPUMS harmonized industry coding based on the 1990 Census Bureau industrial classification scheme. Unemployed persons were to give their most recent occupation, if they had worked in the 5 years preceding the interview, otherwise they were classified as “N/A (not applicable)” or “Last worked 1984 or earlier”.

**S1.2 GSS variables**

Detailed information on all GSS variables are included in the GSS cumulative codebook (Smith et al. 2019). In particular, Appendix A of the codebook provides further details on the sample design and weighting. Appendix P discusses experimental forms, including information on Form 1 (the standard or “x” variant wording) versus Form 2 (the “y” variant wording”). Appendix Q discusses rotation and double sample designs, including information on the sub-samples known as “Ballots”.

*Sexual orientation*. Individuals were asked the following question:

*Which of the following best describes you?*

- *Gay, lesbian, or homosexual*
- *Bisexual*
- *Heterosexual or straight*
- *Don’t know*

This question has been included in the GSS since 2008 in Ballots A, B, and C (i.e., in all sub-samples), but has been asked only in Ballots B and C in 2016 and 2018. Individuals who answered “Gay, lesbian, or homosexual” or “Bisexual” have been coded as “Lesbian”, gay, or bisexual”, while individuals who answered “Heterosexual or straight” have been coded as “Straight”. This variable has been coded as missing for individuals who answered “Don’t know”, who refused to answer the question, or who were not asked the question.

*Spending on environment* combines the following two questions:

*We are faced with many problems in this country, none of which can be solved easily or inexpensively. I'm going to name some of these problems, and for each one I'd like you to tell me whether you think we're spending too much money on it, too little money, or about the right amount. […] are we spending too much, too little, or about the right amount on….*

*improving and protecting the environment?*

*the environment?*

- *Too little*
- *About right*
- *Too much*
- *Don't know*

The two questions are included in Ballots A, B, and C (i.e., in all sub-samples), but they are mutually exclusive since they were used in different questionnaire versions (Form 1 versus Form 2), so no persons got asked both questions. From the answer to these questions, a series of indicator variables has been constructed to record a person’s preference for environmental spending. These variables have been coded as missing for individuals who answered “Don’t know”, who refused to answer the question, or who were not asked the question.

*Spending on green energy*. Individuals were asked the following question:

*We are faced with many problems in this country, none of which can be solved easily or inexpensively. I'm going to name some of these problems, and for each one I'd like you to tell me whether you think we're spending too much money on it, too little money, or about the right amount. […] are we spending too much, too little, or about the right amount on…*

*developing alternative energy sources?*

- *Too little*
- *About right*
- *Too much*
- *Don't know*

This question has been included in the GSS since 2010 in Ballots A, B, and C (i.e., in all sub-samples). From the answer to this question, a series of indicator variables has been constructed to record a person’s preference for green energy spending. These variables have been coded as missing for individuals who answered “Don’t know”, who refused to answer the question, or who were not asked the question.

*Interest in environmental pollution*. Individuals were asked to report their level of interest on issues about environmental pollution. (“Are you very interested, moderately interested, or not at all interested?”). This question has been included in the GSS since 2008. It has been asked in Ballots A, B, and C (i.e., in all sub-samples) in 2008, in Ballots A and C in 2010, in Ballots B and C in 2012 and 2014, and in Ballots A and B in 2016 and 2018. From the answer to this question, a series of indicator variables has been constructed to record a person’s level interest on issues about environmental pollution. These variables have been coded as missing for individuals who answered “Don’t know”, who refused to answer the question, or who were not asked the question.

**Additional references for S1 Text**

Smith, T. W., Davern, M., Freese, J., Morgan, S. L., Son, J., Schapiro, B., & Chatterjee, A. (2019). *General Social Surveys, 1972-2018: Cumulative Codebook*. Chicago, IL.
