# Supplementary material for: Transportation to work by sexual orientation
Source: PLoS One. 2022 Feb 15;17(2):e0263687. doi: 10.1371/journal.pone.0263687 (PMC8846529; doi:10.1371/journal.pone.0263687)
Supplement: S1 Table — Individuals age 18–64 in same-sex and different-sex couples. (DOCX) [file pone.0263687.s002.docx]

**S1 Table. ACS sample sizes. Individuals age 18-64 in same-sex and different-sex couples.**

|  | Individuals in same-sex couples | | Individuals in different-sex couples | |
| --- | --- | --- | --- | --- |
|  | Women | Men | Married | Unmarried |
| 2008 | 5,453 | 5,079 | 997,747 | 96,396 |
| 2009 | 5,703 | 5,285 | 994,337 | 99,090 |
| 2010 | 5,733 | 5,340 | 977,773 | 106,248 |
| 2011 | 5,834 | 5,384 | 945,122 | 104,172 |
| 2012 | 6,080 | 5,603 | 942,970 | 106,056 |
| 2013 | 6,982 | 6,791 | 944,980 | 111,931 |
| 2014 | 7,380 | 7,110 | 929,088 | 113,035 |
| 2015 | 8,061 | 7,723 | 927,944 | 116,554 |
| 2016 | 8,036 | 8,021 | 922,524 | 116,246 |
| 2017 | 8,871 | 8,314 | 926,510 | 121,186 |
| 2018 | 9,137 | 8,975 | 922,169 | 122,709 |
| 2019 | 9,167 | 8,737 | 922,234 | 126,020 |
| Total | 86,437 | 82,362 | 11,353,398 | 1,339,643 |

Notes: Sample includes all respondents (both primary reference person and unmarried partner or married spouse) in a same-sex or different-sex married/unmarried couple. Respondents younger than 18 or older than 64 have been excluded. Source: ACS 2008-2019.
