# Supplementary material for: Transportation to work by sexual orientation
Source: PLoS One. 2022 Feb 15;17(2):e0263687. doi: 10.1371/journal.pone.0263687 (PMC8846529; doi:10.1371/journal.pone.0263687)
Supplement: S2 Table — Individuals age 18–64 in married and unmarried couples. (DOCX) [file pone.0263687.s003.docx]

**S2 Table. ACS sample sizes. Individuals age 18-64 in married and unmarried couples.**

|  | Women in same-sex couples | | Men in same-sex couples | | Individuals in different-sex couples | |
| --- | --- | --- | --- | --- | --- | --- |
|  | Married | Unmarried | Married | Unmarried | Married | Unmarried |
| 2012 | 1,682 | 4,398 | 1,412 | 4,191 | 942,970 | 106,056 |
| 2013 | 2,383 | 4,599 | 2,095 | 4,696 | 944,980 | 111,931 |
| 2014 | 2,909 | 4,471 | 2,891 | 4,219 | 929,088 | 113,035 |
| 2015 | 4,047 | 4,014 | 3,588 | 4,135 | 927,944 | 116,554 |
| 2016 | 4,374 | 3,662 | 4,182 | 3,839 | 922,524 | 116,246 |
| 2017 | 5,296 | 3,575 | 4,681 | 3,633 | 926,510 | 121,186 |
| 2018 | 5,429 | 3,708 | 5,140 | 3,835 | 922,169 | 122,709 |
| 2019 | 5,453 | 3,714 | 4,958 | 3,779 | 922,234 | 126,020 |

Notes: Sample includes all respondents (both primary reference person and unmarried partner or married spouse) in a same-sex or different-sex married/unmarried couple. Respondents younger than 18 or older than 64 have been excluded. Marital status recorded in the ACS for same-sex couples only from 2012. Source: ACS 2012-2019.
