# Supplementary material for: Transportation to work by sexual orientation
Source: PLoS One. 2022 Feb 15;17(2):e0263687. doi: 10.1371/journal.pone.0263687 (PMC8846529; doi:10.1371/journal.pone.0263687)
Supplement: S3 Table — (DOCX) [file pone.0263687.s004.docx]

**S3 Table. Mean comparisons for mode of transportation to work by sex and couple type.**

|  | Women | |  |  | Men | |  |
| --- | --- | --- | --- | --- | --- | --- | --- |
|  | Same-sex couples | Different-sex couples |  |  | Same-sex couples | Different-sex couples |  |
| Variable | (1) | (2) | Gap |  | (3) | (4) | Gap |
| Work from home | 0.053 | 0.058 | -0.004^***^ |  | 0.068 | 0.046 | 0.021^***^ |
| Walk | 0.024 | 0.016 | 0.008^***^ |  | 0.036 | 0.015 | 0.021^***^ |
| Bike | 0.008 | 0.002 | 0.006^***^ |  | 0.011 | 0.005 | 0.006^***^ |
| Public transport | 0.061 | 0.036 | 0.025^***^ |  | 0.101 | 0.035 | 0.066^***^ |
| Taxi | 0.002 | 0.001 | 0.001^***^ |  | 0.003 | 0.001 | 0.002^***^ |
| Drive | 0.844 | 0.882 | -0.038^***^ |  | 0.771 | 0.888 | -0.117^***^ |
| N | 68,403 | 4,343,006 |  |  | 66,059 | 5,144,777 |  |

Weighted means. Sample size (N) refers to the total number of respondents in the relevant sub-group (i.e., those working in the week preceding the ACS interview and who reported their primary means of transportation to work in the preceding week). Respondents younger than 18 or older than 64 have been excluded. Source: ACS 2008-2019. ^*^ *p* < 0.10, ^**^ *p* < 0.05, ^***^ *p* < 0.01
