# Supplementary material for: Transportation to work by sexual orientation
Source: PLoS One. 2022 Feb 15;17(2):e0263687. doi: 10.1371/journal.pone.0263687 (PMC8846529; doi:10.1371/journal.pone.0263687)
Supplement: S4 Table — (DOCX) [file pone.0263687.s005.docx]

**S4 Table. Descriptive statistics by sex and couple type.**

|  | Women | |  | Men | |
| --- | --- | --- | --- | --- | --- |
|  | Same-sex couples | Different-sex couples |  | Same-sex couples | Different-sex couples |
| Variable | (1) | (2) |  | (3) | (4) |
| Age | 42.303 | 44.069 |  | 43.542 | 45.096 |
| White | 0.806 | 0.794 |  | 0.818 | 0.791 |
| Black | 0.095 | 0.074 |  | 0.064 | 0.085 |
| Asian | 0.027 | 0.066 |  | 0.045 | 0.055 |
| Other race | 0.072 | 0.067 |  | 0.073 | 0.069 |
| Hispanic | 0.126 | 0.146 |  | 0.148 | 0.152 |
| Bachelor’s degree | 0.447 | 0.361 |  | 0.488 | 0.340 |
| Has child | 0.326 | 0.599 |  | 0.137 | 0.621 |
| Has child age 0-4 | 0.088 | 0.188 |  | 0.043 | 0.201 |
| Married | 0.483 | 0.876 |  | 0.460 | 0.870 |
| Student | 0.095 | 0.061 |  | 0.076 | 0.041 |
| In the army | 0.004 | 0.001 |  | 0.003 | 0.009 |
| Employed | 0.810 | 0.680 |  | 0.821 | 0.858 |
| In the labor force | 0.849 | 0.715 |  | 0.860 | 0.895 |
| Total family income | 74,282 | 101,340 |  | 101,851 | 101,117 |
| N | 86,437 | 6,554,055 |  | 82,362 | 6,138,986 |

Weighted means. Sample size (N) refers to the total number of respondents in the relevant sub-group (i.e., individuals in same-sex or different-sex couples). Respondents younger than 18 or older than 64 have been excluded. Source: ACS 2008-2019 (2012-2019 for marital status). All differences are statistically significant at the 1-percent level.
