# Supplementary material for: Transportation to work by sexual orientation
Source: PLoS One. 2022 Feb 15;17(2):e0263687. doi: 10.1371/journal.pone.0263687 (PMC8846529; doi:10.1371/journal.pone.0263687)
Supplement: S5 Table — By sex and couple type. Additional restrictions. (DOCX) [file pone.0263687.s006.docx]

**S5 Table. Drive to work. By sex and couple type. Additional restrictions.**

|  | No students | No army | 2012-2019 | Cluster SE | No weights | Logit |
| --- | --- | --- | --- | --- | --- | --- |
|  | (1) | (2) | (3) | (4) | (5) | (6) |
| *Panel A: Women in SSC and DSC* |  |  |  |  |  |  |
| In a same-sex couple | -0.018^***^ | -0.020^***^ | -0.024^***^ | -0.020^***^ | -0.021^***^ | -0.017^***^ |
|  | (0.002) | (0.002) | (0.002) | (0.002) | (0.001) | (0.002) |
| Observations | 4,139,712 | 4,404,566 | 2,940,098 | 4,411,409 | 4,411,409 | 4,411,409 |
| Mean of dependent variable | 0.880 | 0.881 | 0.877 | 0.881 | 0.881 | 0.881 |
| R^2^ | 0.046 | 0.047 | 0.048 | 0.047 | 0.039 | - |
| Pseudo R^2^ | - | - | - | - | - | 0.055 |
|  |  |  |  |  |  |  |
| *Panel B: Men in SSC and DSC* |  |  |  |  |  |  |
| In a same-sex couple | -0.073^***^ | -0.072^***^ | -0.075^***^ | -0.072^***^ | -0.072^***^ | -0.053^***^ |
|  | (0.002) | (0.002) | (0.002) | (0.002) | (0.002) | (0.002) |
| Observations | 5,007,438 | 5,154,713 | 3,467,410 | 5,210,836 | 5,210,836 | 5,210,836 |
| Mean of dependent variable | 0.887 | 0.886 | 0.884 | 0.887 | 0.887 | 0.887 |
| R^2^ | 0.052 | 0.053 | 0.055 | 0.052 | 0.046 | - |
| Pseudo R^2^ | - | - | - | - | - | 0.063 |
|  |  |  |  |  |  |  |
| *Controls for:* |  |  |  |  |  |  |
| State and year FE | 🗸 | 🗸 | 🗸 | 🗸 | 🗸 | 🗸 |
| Demographic controls | 🗸 | 🗸 | 🗸 | 🗸 | 🗸 | 🗸 |
| Partner/spouse controls | 🗸 | 🗸 | 🗸 | 🗸 | 🗸 | 🗸 |
| Fertility and marital status | 🗸 | 🗸 | 🗸 | 🗸 | 🗸 | 🗸 |

See also notes in Table 1. Source: ACS 2008-2019 (2012-2019 in Column 3). ^*^ *p* < 0.10, ^**^ *p* < 0.05, ^***^ *p* < 0.01.
