# Supplementary material for: Transportation to work by sexual orientation
Source: PLoS One. 2022 Feb 15;17(2):e0263687. doi: 10.1371/journal.pone.0263687 (PMC8846529; doi:10.1371/journal.pone.0263687)
Supplement: S6 Table — By sex, couple type, and age group. (DOCX) [file pone.0263687.s007.docx]

**S6 Table. Drive to work. By sex, couple type, and age group.**

|  | 18-40 | 41-64 | 25-64 |
| --- | --- | --- | --- |
|  | (1) | (2) | (3) |
| *Panel A: Women in SSC and DSC* |  |  |  |
| In a same-sex couple | -0.022^***^ | -0.020^***^ | -0.018^***^ |
|  | (0.003) | (0.002) | (0.002) |
| Observations | 1,646,346 | 2,765,063 | 4,258,026 |
| Mean of dependent variable | 0.880 | 0.882 | 0.880 |
| R^2^ | 0.069 | 0.034 | 0.046 |
|  |  |  |  |
| *Panel B: Men in SSC and DSC* |  |  |  |
| In a same-sex couple | -0.076^***^ | -0.071^***^ | -0.072^***^ |
|  | (0.003) | (0.002) | (0.002) |
| Observations | 1,852,711 | 3,358,125 | 5,090,197 |
| Mean of dependent variable | 0.888 | 0.886 | 0.886 |
| R^2^ | 0.076 | 0.041 | 0.052 |
|  |  |  |  |
| *Controls for:* |  |  |  |
| State and year FE | 🗸 | 🗸 | 🗸 |
| Demographic controls | 🗸 | 🗸 | 🗸 |
| Partner/spouse controls | 🗸 | 🗸 | 🗸 |
| Fertility and marital status | 🗸 | 🗸 | 🗸 |

See also notes in Table 1. Source: ACS 2008-2019. ^*^ *p* < 0.10, ^**^ *p* < 0.05, ^***^ *p* < 0.01.
