# Supplementary material for: Transportation to work by sexual orientation
Source: PLoS One. 2022 Feb 15;17(2):e0263687. doi: 10.1371/journal.pone.0263687 (PMC8846529; doi:10.1371/journal.pone.0263687)
Supplement: S7 Table — By sex, couple type, race, and ethnicity. (DOCX) [file pone.0263687.s008.docx]

**S7 Table. Drive to work. By sex, couple type, race, and ethnicity.**

|  | White | Black | Asian | Hispanic |
| --- | --- | --- | --- | --- |
|  | (1) | (2) | (3) | (4) |
| *Panel A: Women in SSC and DSC* |  |  |  |  |
| In a same-sex couple | -0.021^***^ | -0.018^***^ | -0.002 | 0.0004 |
|  | (0.002) | (0.007) | (0.011) | (0.0048) |
| Observations | 3,650,782 | 268,726 | 261,460 | 469,924 |
| Mean of dependent variable | 0.890 | 0.869 | 0.822 | 0.864 |
| R^2^ | 0.032 | 0.117 | 0.121 | 0.097 |
|  |  |  |  |  |
| *Panel B: Men in SSC and DSC* |  |  |  |  |
| In a same-sex couple | -0.073^***^ | -0.068^***^ | -0.075^***^ | -0.064^***^ |
|  | (0.002) | (0.009) | (0.010) | (0.005) |
| Observations | 4,313,957 | 310,190 | 281,674 | 649,412 |
| Mean of dependent variable | 0.892 | 0.884 | 0.837 | 0.893 |
| R^2^ | 0.044 | 0.088 | 0.106 | 0.096 |
|  |  |  |  |  |
| *Controls for:* |  |  |  |  |
| State and year FE | 🗸 | 🗸 | 🗸 | 🗸 |
| Demographic controls | 🗸 | 🗸 | 🗸 | 🗸 |
| Partner/spouse controls | 🗸 | 🗸 | 🗸 | 🗸 |
| Fertility and marital status | 🗸 | 🗸 | 🗸 | 🗸 |

See also notes in Table 1. Demographic controls in these specifications include only age and education, not race or ethnicity. Source: ACS 2008-2019. ^*^ *p* < 0.10, ^**^ *p* < 0.05, ^***^ *p* < 0.01.
