# Supplementary material for: Transportation to work by sexual orientation
Source: PLoS One. 2022 Feb 15;17(2):e0263687. doi: 10.1371/journal.pone.0263687 (PMC8846529; doi:10.1371/journal.pone.0263687)
Supplement: S8 Table — By sex, couple type, marital status, and fertility. (DOCX) [file pone.0263687.s009.docx]

**S8 Table. Drive to work. By sex, couple type, marital status, and fertility.**

|  | Only married | Only unmarried | With children | Without children |
| --- | --- | --- | --- | --- |
|  | (1) | (2) | (3) | (4) |
| *Panel A: Women in SSC and DSC* |  |  |  |  |
| In a same-sex couple | -0.052^***^ | 0.007^***^ | -0.010^***^ | -0.019^***^ |
|  | (0.003) | (0.003) | (0.003) | (0.002) |
| Observations | 2,568,246 | 371,852 | 2,501,737 | 1,909,672 |
| Mean of dependent variable | 0.878 | 0.870 | 0.886 | 0.876 |
| R^2^ | 0.044 | 0.080 | 0.040 | 0.057 |
|  |  |  |  |  |
| *Panel B: Men in SSC and DSC* |  |  |  |  |
| In a same-sex couple | -0.079^***^ | -0.063^***^ | -0.035^***^ | -0.067^***^ |
|  | (0.003) | (0.003) | (0.005) | (0.002) |
| Observations | 3,065,003 | 402,407 | 3,226,899 | 1,983,937 |
| Mean of dependent variable | 0.886 | 0.866 | 0.895 | 0.873 |
| R^2^ | 0.051 | 0.083 | 0.047 | 0.062 |
|  |  |  |  |  |
| *Controls for:* |  |  |  |  |
| State and year FE | 🗸 | 🗸 | 🗸 | 🗸 |
| Demographic controls | 🗸 | 🗸 | 🗸 | 🗸 |
| Partner/spouse controls | 🗸 | 🗸 | 🗸 | 🗸 |
| Fertility | 🗸 | 🗸 |  |  |
| Marital status |  |  | 🗸 | 🗸 |

See also notes in Table 1. Source: ACS 2008-2019 (2012-2019 in Columns 1-2). ^*^ *p* < 0.10, ^**^ *p* < 0.05, ^***^ *p* < 0.01.
