# Supplementary material for: Transportation to work by sexual orientation
Source: PLoS One. 2022 Feb 15;17(2):e0263687. doi: 10.1371/journal.pone.0263687 (PMC8846529; doi:10.1371/journal.pone.0263687)
Supplement: S9 Table — By sex, couple type, and position in household. (DOCX) [file pone.0263687.s010.docx]

**S9 Table. Drive to work. By sex, couple type, and position in household.**

|  | Household head | Spouse or partner | Main earner |
| --- | --- | --- | --- |
|  | (1) | (2) | (3) |
| *Panel A: Women in SSC and DSC* |  |  |  |
| In a same-sex couple | -0.014^***^ | -0.024^***^ | -0.013^***^ |
|  | (0.002) | (0.002) | (0.002) |
| Observations | 1,846,540 | 2,564,869 | 1,753,489 |
| Mean of dependent variable | 0.873 | 0.888 | 0.880 |
| R^2^ | 0.047 | 0.047 | 0.062 |
|  |  |  |  |
| *Panel B: Men in SSC and DSC* |  |  |  |
| In a same-sex couple | -0.074^***^ | -0.071^***^ | -0.071^***^ |
|  | (0.003) | (0.003) | (0.002) |
| Observations | 3,173,588 | 2,037,248 | 4,004,896 |
| Mean of dependent variable | 0.885 | 0.890 | 0.887 |
| R^2^ | 0.053 | 0.052 | 0.054 |
|  |  |  |  |
| *Controls for:* |  |  |  |
| State and year FE | 🗸 | 🗸 | 🗸 |
| Demographic controls | 🗸 | 🗸 | 🗸 |
| Partner/spouse controls | 🗸 | 🗸 | 🗸 |
| Fertility and marital status | 🗸 | 🗸 | 🗸 |

See also notes in Table 1. Source: ACS 2008-2019. ^*^ *p* < 0.10, ^**^ *p* < 0.05, ^***^ *p* < 0.01.
