# Supplementary material for: Transportation to work by sexual orientation
Source: PLoS One. 2022 Feb 15;17(2):e0263687. doi: 10.1371/journal.pone.0263687 (PMC8846529; doi:10.1371/journal.pone.0263687)
Supplement: S10 Table — By sex and couple type. Additional subsamples. (DOCX) [file pone.0263687.s011.docx]

**S10 Table. Drive to work. By sex and couple type. Additional subsamples.**

|  | Only students | Only army | Both working |
| --- | --- | --- | --- |
|  | (1) | (2) | (3) |
| *Panel A: Women in SSC and DSC* |  |  |  |
| In a same-sex couple | -0.037^***^ | -0.068^***^ | -0.020^***^ |
|  | (0.005) | (0.021) | (0.002) |
| Observations | 271,697 | 6,843 | 3,702,772 |
| Mean of dependent variable | 0.903 | 0.946 | 0.882 |
| R^2^ | 0.054 | 0.046 | 0.046 |
|  |  |  |  |
| *Panel B: Men in SSC and DSC* |  |  |  |
| In a same-sex couple | -0.064^***^ | -0.046^**^ | -0.074^***^ |
|  | (0.007) | (0.023) | (0.002) |
| Observations | 203,398 | 56,123 | 3,613,617 |
| Mean of dependent variable | 0.890 | 0.943 | 0.890 |
| R^2^ | 0.064 | 0.020 | 0.053 |
|  |  |  |  |
| *Controls for:* |  |  |  |
| State and year FE | 🗸 | 🗸 | 🗸 |
| Demographic controls | 🗸 | 🗸 | 🗸 |
| Partner/spouse controls | 🗸 | 🗸 | 🗸 |
| Fertility and marital status | 🗸 | 🗸 | 🗸 |

See also notes in Table 1. Source: ACS 2008-2019. ^*^ *p* < 0.10, ^**^ *p* < 0.05, ^***^ *p* < 0.01.
