# Supplementary material for: Transportation to work by sexual orientation
Source: PLoS One. 2022 Feb 15;17(2):e0263687. doi: 10.1371/journal.pone.0263687 (PMC8846529; doi:10.1371/journal.pone.0263687)
Supplement: S11 Table — By sex and couple type. (DOCX) [file pone.0263687.s012.docx]

**S11 Table. Multinomial logit** **of type of transport to work (driving, public, or active). By sex and couple type.**

|  | (1) | (2) | (3) | (4) | (5) |
| --- | --- | --- | --- | --- | --- |
| *Panel A: Women in SSC and DSC* |  |  |  |  |  |
|  |  |  |  |  |  |
| *Public transport* |  |  |  |  |  |
| In a same-sex couple | 1.795^***^ | 1.725^***^ | 1.752^***^ | 1.713^***^ | 1.285^***^ |
|  | (0.035) | (0.036) | (0.038) | (0.037) | (0.029) |
| *Active transport* |  |  |  |  |  |
| In a same-sex couple | 1.811^***^ | 1.745^***^ | 1.749^***^ | 1.717^***^ | 1.292^***^ |
|  | (0.048) | (0.047) | (0.047) | (0.047) | (0.037) |
| Observations | 4,129,831 | 4,129,831 | 4,129,831 | 4,129,831 | 4,129,831 |
| Pseudo R^2^ | 0.001 | 0.124 | 0.150 | 0.153 | 0.160 |
|  |  |  |  |  |  |
| *Panel B: Men in SSC and DSC* |  |  |  |  |  |
|  |  |  |  |  |  |
| *Public transport* |  |  |  |  |  |
| In a same-sex couple | 3.332^***^ | 2.906^***^ | 2.682^***^ | 2.758^***^ | 2.053^***^ |
|  | (0.053) | (0.051) | (0.049) | (0.050) | (0.039) |
| *Active transport* |  |  |  |  |  |
| In a same-sex couple | 2.628^***^ | 2.408^***^ | 2.240^***^ | 2.280^***^ | 1.453^***^ |
|  | (0.023) | (0.057) | (0.053) | (0.054) | (0.036) |
| Observations | 4,924,212 | 4,924,212 | 4,924,212 | 4,924,212 | 4,924,212 |
| Pseudo R^2^ | 0.003 | 0.123 | 0.147 | 0.150 | 0.154 |
|  |  |  |  |  |  |
| *Controls for:* |  |  |  |  |  |
| State and year FE |  | 🗸 | 🗸 | 🗸 | 🗸 |
| Demographic controls |  |  | 🗸 | 🗸 | 🗸 |
| Partner/spouse controls |  |  |  | 🗸 | 🗸 |
| Fertility and marital status |  |  |  |  | 🗸 |

See also notes in Table 1. Source: ACS 2008-2019. ^*^ *p* < 0.10, ^**^ *p* < 0.05, ^***^ *p* < 0.01. Relative risk ratios reported, with driving to work as base outcome. Active transport is defined as walking or biking to work.
