# Supplementary material for: Transportation to work by sexual orientation
Source: PLoS One. 2022 Feb 15;17(2):e0263687. doi: 10.1371/journal.pone.0263687 (PMC8846529; doi:10.1371/journal.pone.0263687)
Supplement: S12 Table — Individuals age 18–64 by sexual orientation. (DOCX) [file pone.0263687.s013.docx]

**S12 Table. GSS sample sizes. Individuals age 18-64 by sexual orientation.**

|  | Gay, lesbian,  or homosexual | Bisexual | Heterosexual  or straight | Don’t know | No answer | Not applicable |
| --- | --- | --- | --- | --- | --- | --- |
| 2008 | 32 | 26 | 1,398 | 5 | 18 | 159 |
| 2010 | 27 | 31 | 1,436 | 7 | 14 | 133 |
| 2012 | 24 | 40 | 1,345 | 2 | 22 | 142 |
| 2014 | 38 | 60 | 1,770 | 5 | 29 | 109 |
| 2016 | 43 | 52 | 1,318 | 3 | 21 | 803 |
| 2018 | 29 | 47 | 1,020 | 4 | 22 | 680 |
| Total | 193 | 256 | 8,287 | 26 | 126 | 2,026 |

Notes: Respondents younger than 18 or older than 64 have been excluded. “Not applicable” includes respondents who were not asked the relevant question. Source: GSS 2008-2018.
