# Supplementary material for: Transportation to work by sexual orientation
Source: PLoS One. 2022 Feb 15;17(2):e0263687. doi: 10.1371/journal.pone.0263687 (PMC8846529; doi:10.1371/journal.pone.0263687)
Supplement: S13 Table — (DOCX) [file pone.0263687.s014.docx]

**S13 Table. Mean comparisons for environmental preferences by sexual orientation.**

|  | GLB | Straight |  |
| --- | --- | --- | --- |
| Variable | (1) | (2) | Gap |
| *Improving and protecting environment* |  |  |  |
| Spending too little | 0.731 | 0.656 | 0.075^***^ |
| Spending about right | 0.197 | 0.257 | -0.060^***^ |
| Spending too much | 0.072 | 0.087 | -0.015 |
| *Developing alternative energy sources* |  |  |  |
| Spending too little | 0.707 | 0.603 | 0.104^***^ |
| Spending about right | 0.208 | 0.313 | -0.105^***^ |
| Spending too much | 0.085 | 0.084 | 0.001 |
| *Interested in issues on environmental pollution* |  |  |  |
| Very interested | 0.542 | 0.454 | 0.088^**^ |
| Moderately interested | 0.382 | 0.455 | -0.073^*^ |
| Not at all interested | 0.076 | 0.091 | -0.014 |
| N | 449 | 8,287 |  |

Weighted means. “GLB” refers to individual who identifies as gay, lesbian, homosexual, or bisexual when asked about their sexual orientation. Sample size (N) refers to the total number of respondents in the relevant sub-group. Respondents younger than 18 or older than 64 have been excluded. Respondents who answered “don’t know”, who refused to answer, or who were not asked the relevant questions are not included in the above comparisons. Source: GSS 2008-2018. ^*^ *p* < 0.10, ^**^ *p* < 0.05, ^***^ *p* < 0.01
